# 2087: Better Together Lebanon County: A collaboration to improve the health environment and reduce obesity through community-owned initiatives

**DOI:** 10.1017/cts.2017.243

**Published:** 2018-05-10

**Authors:** Erica Francis, Brianna Hoglen, Kara Shifler, Jennifer Kraschnewski, Jeanne Donlevy Arnold, Ruth Ellen Hogentogler, Pamela Witt

## Abstract

OBJECTIVES/SPECIFIC AIMS: Improving public health requires effective community-engaged approaches. The Better Together Lebanon County initiative plans to create opportunities for improved health and quality of life by aligning strategies of local organizations, previously working independently. METHODS/STUDY POPULATION: Better Together began with a 1-day summit, convening stakeholders with the goal of coordinating efforts and maximizing resources in the Lebanon community. Key stakeholders were identified using the socioecological model to assist with planning, goal setting, and developing outcomes for this initiative. Representation included community members, hospital systems, restaurants, school administrators, nonprofit organizations (including YMCA, American Heart Association), grocery stores and policy makers such as the mayor, health departments, and state representatives. RESULTS/ANTICIPATED RESULTS: The Better Together summit brought together almost 200 individuals representing 82 local organizations to share ideas and evoke collaboration around decreasing health disparities. Attendees learned about programs within and outside of their communities and volunteered for task forces to propel the community forward. Currently, we have members committed to further this work through Action Teams within the sectors of Physical Activity, Healthy Food Access and Family and Community Engagement. DISCUSSION/SIGNIFICANCE OF IMPACT: Convening individuals from many layers of the community helps to ensure discussions are representative of the overall community voice. It is vital to facilitate effective collaboration that includes networking, identifying assets and areas of improvement, brainstorming solutions and integrating research and best practices to improve the health of a community.

